# The path to fertility: Current approaches to mare endometritis and endometrosis

**DOI:** 10.1590/1984-3143-AR2024-0070

**Published:** 2024-09-13

**Authors:** Graça ML Ferreira-Dias, Joana Alpoim-Moreira, Anna Szóstek-Mioduchowska, Maria Rosa Rebordão, Dariusz J. Skarzynski

**Affiliations:** 1 Centro de Investigação Interdisciplinar em Sanidade Animal, Faculdade de Medicina Veterinária, Universidade de Lisboa, Lisboa, Portugal; 2 AL4AnimalS-Associate Laboratory for Animal and Veterinary Sciences, Lisboa, Portugal; 3 Institute of Animal Reproduction and Food Research PAS, Olsztyn, Poland; 4 Research Center for Natural Resources, Environment and Society, Polytechnic University of Coimbra, Coimbra, Portugal; 5 Polytechnic University of Coimbra, S. Martinho do Bispo, Coimbra, Portugal; 6 Department of Animal Reproduction with Large Animal Clinic, Faculty of Veterinary Medicine, University of Environmental and Live Sciences, Wrocław, Poland

**Keywords:** mare, endometritis, endometrosis, Neutrophil Extracellular Traps, epigenetics

## Abstract

The path to fertility in the mare requires an understanding of the hormonal influences, the immune response, genetics, and epigenetic mechanisms involved not only in physiological reproductive processes, but also such pathologies as endometritis and endometrosis. Endometritis may lead to endometrosis establishment. In the presence of endometritis, neutrophils arrive at the mare endometrium, and form neutrophil extracellular traps. While NETosis plays pivotal roles, prolonged inflammation can lead to chronic endometritis, endometrosis, and fertility issues. Matrix metalloproteinases and epigenetic changes influence the course of endometrosis. Inhibitors of specific enzymes involved in NETosis and epigenetic inhibitors have shown potential in reducing pro-fibrotic effects. Collagen type III (COL3) has emerged as a putative biomarker, correlating with endometrosis and useful in fertility assessment. Thus, COL3 may offer a non-invasive diagnostic tool, as a complement to histopathological methods. Epigenetic modifications and miRNA expressions offer new avenues for therapeutic strategies, emphasizing the importance of understanding the cellular mechanisms at play in mare endometrial fibrosis.

## Introduction

Endometritis is an inflammatory condition in mares that occurs as a physiological response to breeding. Failure to resolve this inflammation can lead to persistent breeding-induced endometritis, which if untreated can lead to endometrial fibrosis, ultimately resulting in early embryonic death and reduced fertility. Following breeding, neutrophils are the first line of defense of the immune system (Katila and Ferreira-Dias, 2022). These cells initiate the inflammatory response that clears pathogens through phagocytosis, extracellular lytic functions, and by NETosis. NETosis involves the release of nuclear contents to form neutrophil extracellular traps (NETs), which capture and neutralize pathogens (Brinkmann et al., 2004). NETs are composed of DNA fibers and proteins, like histones, elastase (ELA), cathepsin G (CAT), and myeloperoxidase (MPO) that can capture and kill pathogens. We have shown that equine neutrophils release NETs in vitro and ex vivo in response to bacterial strains responsible for mare endometritis. This suggests a complementary mechanism to combat bacteria involved in endometritis in mares (Rebordão et al., 2014). However, while NETs have a strong antibacterial effect prolonged NETosis can contribute to mare endometrial fibrogenesis (Rebordão et al., 2018). The pro-fibrotic effect of NETs appears to depend on uterine endocrine control mechanisms with the follicular phase favoring collagen (COL) production. Such differences may be mediated by tissue-specific catabolic or anabolic enzymes involved in COL synthesis (Rebordão et al., 2018). Also, NETs enzymes (ELA and CAT) induce high production of PGF_2_α and/or PTGFR transcription in mare endometrium, potentially facilitating fibrogenesis (Rebordão et al., 2021). In vitro studies indicate that impaired PGE_2_ production and transcript reduction of one of the four prostanoid subtypes receptors (EP2 receptor), may be associated with endometrial fibrogenesis (Rebordão et al., 2019).

## Extracellular Matrix regulation

A well-controlled balance between the function of matrix metalloproteinases (MMPs) and their inhibitors (TIMPs) is crucial in maintaining the extracellular matrix (ECM). In the endometrium of mares with mild to moderate lesions, there is an upregulation of MMP-2 and MMP-9, with increased MMP-9 in TGFβ1-treated fibroblasts and epithelial cells (Szóstek-Mioduchowska et al., 2020a). We have shown that growth factor TGF-β1, interleukins (IL-6, IL-1β) and prostaglandins (PGE_2_, PGF_2α_) affect the expression of ECM-associated genes and proteins, mare endometrium fibroblast proliferation and myofibroblast differentiation (Szóstek et al., 2014; Szóstek-Mioduchowska et al., 2019a, b, 2020a, b). These processes are associated with endometrosis development and link inflammation with fibrotic processes. In addition, the response of endometrial tissue or fibroblasts differs, depending on the severity of endometrosis (Szóstek-Mioduchowska et al., 2019b, 2023; Wójtowicz et al., 2023).

## Protease inhibitors as putative therapeutic agents

Our recent studies explored the role of specific protease inhibitors in mitigating the effects of enzymes, which are implicated in the pro-fibrotic state associated with NETs persistence. As such, the use of NETs specific proteases inhibitors (ELA- Sivelestat; MPO- 4-aminobenzoic acid hydrazide; CAT- β-keto-phosphonic acid) or a non-specific protease inhibitor, Noscapine, reverted the in vitro pro-fibrotic effects of those proteases in equine endometrium (Amaral et al., 2018, 2020a, b, 2021a, b, c, 2023). ELA inhibition stimulated in vitro production of anti-fibrotic PGE_2_, and inhibited the profibrotic PGF_2α_ (Amaral et al., 2020a), while CAT and MPO inhibition reversed the CAT- and MPO-induce activity of MMP-2/MMP-9 (Amaral et al., 2020b, 2021a). Therefore, NETs protease inhibition can be a potential profilactic or therapeutic approach to endometrosis.

## Biomarkers for diagnosis and prognosis of fertility

The gold standard method for endometrosis evaluation has been endometrial biopsy histopathological classification. Currently, the fertility prognosis is based on the categorization scales of Kenney and Doig (1986) and Schoon et al. (1997). Even though it has been considered as a safe and useful method, the search for a less invasive technique is desirable. Our previous work has shown that serum COL type 3 (COL3) may prove useful as a diagnostic aid of endometrosis, and as a fertility indicator, since it was positively correlated with infertility (Alpoim-Moreira et al., 2022a). It may be used to evaluate recipient mares in embryo transfer programs, when performing endometrial biopsies is not practicable. Thus, COL3 may offer a non-invasive diagnostic tool, as a complement to histopathological methods.

## Epigenetics and genomics in endometrium

DNA methylation is responsible for gene control and is one of many epigenetic mechanisms in fibroproliferative diseases. DNA hypermethylation is usually associated with gene repression, and hypomethylation with increased gene expression. This stable epigenetic marker can be assessed through DNA methyltransferases (DNMTs) action and/or by methylation analysis of a particular gene zone through bisulfite pyrosequencing. Since epigenetic changes can be reversed, they may be used as therapeutic targets. We conducted studies to assess the epigenetic involvement in mare endometrosis. It was observed that *DNMT3B mRNA* transcripts and COL1 and COL3 protein expression increased in fibrotic category III endometrium, when compared to category I, pointing to an epigenetic role (Alpoim-Moreira et al., 2019). Bisulfite pyrosequencing of promoter or regulatory regions of *MMP2* and *MMP9* genes, detected hypermethylation of those regions, and mRNA levels decrease, as endometrosis progressed. Thus, hypermethylation might be responsible for repressing their transcription. Transcription inhibition of *MMP2* and *MMP9* gene expression, resulting from hypermethylation of their promoter and regulatory regions, may lead to a diminished COL degradation and endometrium accumulation in advanced endometrosis (Alpoim-Moreira et al., 2022b).

Since fibroblasts have a crucial role in fibrogenesis, mare endometrial TGF-β1 treated fibroblasts showed increased *DNMT3A* transcripts, COL1 and COL3 transcripts and protein expression, and decreased *MMP2* mRNA and activity. This was reduced by the epigenetic inhibitor decitabine. Bisulfite pyrosequencing of the promoter or regulatory regions of *COL1A1* and *MMP9* genes showed an hypermethylation in *MMP9* after TGF-β1 treated fibroblasts. That hypermethylation decreased by decitabine addition. Epigenetic modulation may occur through DNA hypermethylation, via anti-fibrotic genes rather than fibrotic genes (Alpoim-Moreira et al., 2023).

Next-generation sequencing analysis of mare endometrium allowed the identification of potential pathways and regulators involved in endometrosis development (Szóstek-Mioduchowska et al., 2023;Wójtowicz et al., 2023). The functional enrichment obtained from the transcriptomic data suggests that inflammation and metabolic changes may be features of categories IIA and IIB endometrium. Differentially expressed genes (DEGs) were annotated to inflammation, cellular infiltration by leukocytes, macrophages, and phagocytes; and cytokine quantity in mild (IIA) and moderate (IIB) categories vs. category I endometrium. These data together with our previous results (Szóstek et al., 2014; Szóstek-Mioduchowska et al., 2019a, b, 2020a, b) confirmed findings on fibrogenesis in many organs of other species. This suggests that pro-inflammatory cytokines and growth factors regulate fibrosis, either indirectly by attracting inflammatory cells to the site of inflammation, or by acting directly on various tissues.

Endometrial transcriptome analysis in endometrosis suggests changes in gene expression related to cellular metabolism and molecular transport of lipids, carbohydrates, and amino acids, particularly in category IIA vs. category I endometrium. In category IIB vs I, DEGs were linked to mitochondrial dysfunction and oxidative phosphorylation (Szóstek-Mioduchowska et al., 2023).

A growing body of evidence supports the importance of microRNA (miRNA) in fibrosis, and also in mare endometrosis development (Wójtowicz et al., 2023). Our results showed that 1, 26 and 5 miRNAs were differentially expressed (DEmiRs) in category IIA, IIB and III vs. category I endometria, respectively. Therefore, miRNAs might play a role in the moderate stage of endometrosis. These DEmiRs may be associated with fibrosis via their target ECM-associated genes, including COLs, fibronectin (FN), elastin, laminin, MMPs and TIMPs. DEmiRs can also regulate the immune response by affecting interferons, interleukins and their receptors, and interleukin-4-induced gene-1.

In conclusion, understanding the complex interplay of immune responses, molecular pathways, and genetic and epigenetic factors in endometritis and endometrosis provides a foundation for developing diagnostic tools and targeted therapies. Continued research is essential for refining these approaches and improving the overall reproductive health and success of mares.
